# Regulation of Helicobacter pylori Urease and Acetone Carboxylase Genes by Nitric Oxide and the CrdRS Two-Component System

**DOI:** 10.1128/spectrum.04633-22

**Published:** 2023-01-10

**Authors:** Madison G. McKinsey, Miranda Y. Bate, Lauren M. Tramonte, Emely Y. Avalos, John Loh, Timothy L. Cover, Mark H. Forsyth

**Affiliations:** a Department of Biology, College of William & Mary, Williamsburg, Virginia, USA; b Department of Medicine, Vanderbilt University School of Medicine, Nashville, Tennessee, USA; c Department of Pathology, Microbiology, and Immunology, Vanderbilt University Medical Center, Nashville, Tennessee, USA; d Vanderbilt Institute for Infection, Immunology, and Inflammation, Vanderbilt University Medical Center, Nashville, Tennessee, USA; e Veterans Affairs Tennessee Valley Healthcare System, Nashville, Tennessee, USA; South China Sea Institute of Oceanology

**Keywords:** *Helicobacter*, gene regulation, two-component system, urease

## Abstract

Helicobacter pylori colonizes the human gastric mucosa and causes various gastroduodenal diseases, including peptic ulceration and gastric cancer. Colonization requires the actions of two-component systems (TCSs) to sense and respond to changes in the host environment. In this study, we evaluated gene regulation mediated by the CrdRS TCS. Few studies have evaluated this TCS, leaving the signal(s) yet to be exhaustively determined and a need for a more complete regulon to be delineated. We performed RNA sequencing (RNA-Seq) on three isogenic H. pylori 26695 mutants: a control, a mutant with deletion of the sensory histidine kinase, Δ*crdS*, and a mutant with deletion of the response regulator, Δ*crdR*. Comparison of the RNA-Seq results from these mutants established a 40-gene regulon putatively controlled by the CrdRS TCS. Quantitative reverse transcriptase PCR (RT-qPCR) was used to validate 7 of 11 putative regulon members selected for analysis. We further investigated 6 confirmed CrdRS regulon genes by using phospho-incompetent H. pylori 26695 CrdR D53A and CrdS H173A mutants. These experiments further confirmed the role of CrdRS in regulation of urease, acetone carboxylase, *hofD*, and *HP1440.* Expression of these CrdRS regulon genes was also evaluated under 10 μM nitric oxide (NO) conditions. This revealed that *ureA*, *acxA*, *hofD*, and *HP1440* expression is affected by NO in a CrdRS-dependent manner. Importantly, three of these genes (*ureA*, *acxA*, and *hofD*) are known to play important roles in H. pylori colonization of the stomach.

**IMPORTANCE** The molecular strategies used by Helicobacter pylori to colonize and persist in the harsh environment of the human stomach are a critical area of study. Our study identified several genes in this gastric pathogen, including *ureA*, a gene encoding a protein essential to the survival of H. pylori, that are regulated via the CrdRS two-component system (TCS) in response to nitric oxide (NO). NO is a product of the innate immune system of the human host. The identification of these genes whose expression is regulated by this molecule may give insights to novel therapeutics. Two genes (*ureA* and *acxA*) determined in this study to be regulated by NO via CrdRS have been previously determined to be regulated by other TCSs, indicating that the expression of these genes may be of critical importance to H. pylori.

## INTRODUCTION

Helicobacter pylori is a Gram-negative bacterium that colonizes the human gastric mucosa (1). It was the first formally recognized bacterial class I carcinogen and is one of the most successful human pathogens. H. pylori infects humans globally, but infection rates vary significantly from nation to nation (2), and infection is associated with various diseases, including duodenal and gastric ulcers, premalignant lesions (atrophic gastritis and intestinal metaplasia), and gastric adenocarcinoma (3–6). H. pylori-associated gastric cancer is one of the leading causes of cancer death worldwide (7).

H. pylori’s ability to cause various disease manifestations including cancer is related to its ability to colonize and persist long-term within the human stomach, a harsh environment due to its acidic pH. H. pylori has several mechanisms that promote successful colonization, including use of the enzyme urease to mitigate gastric acidity. Urease hydrolyzes urea to yield ammonia and carbon dioxide, creating a more neutral periplasm and adjacent external environment (8, 9).

In response to H. pylori colonization, the host immune system is activated. H. pylori mitigates recognition by the innate immune response by modulation of its surface lipopolysaccharide and flagellin to evade host detection (10). When the innate and adaptive immune responses are successfully initiated despite H. pylori’s avoidance strategies, H. pylori is challenged by a toxic environment characterized by the production of reactive oxygen species (ROS), such as hydrogen peroxide and superoxide anions, and reactive nitrogen species (RNS), most notably nitric oxide (NO) (11).

Two-component systems (TCSs) are signal transduction pathways that allow a bacterium to sense and respond to external stimuli in its environment, including the RNS elicited during the human immune response (12). TCSs are the primary means by which bacteria alter gene expression to acclimate to changing environmental conditions (13). In addition to the CheAY chemotaxis system, H. pylori encodes three complete TCSs: ArsRS, FlgRS, and CrdRS (14–17). These TCSs facilitate H. pylori infection, adaptation, and persistence within the human stomach (12, 18–20).

The ArsRS TCS is involved in acidic acclimation. It ensures successful colonization of the gastric mucosa by H. pylori (21, 22). The ArsRS TCS regulates the expression of multiple genes, including components of the urease system, which mitigate the low-pH stress immediately surrounding the bacterium (16, 23, 24). The FlgRS TCS is known to activate the transcription of σ^54^ in H. pylori, thus facilitating expression of flagellar structural genes (15). The use of flagella in gastric mucus layer penetration is also a critical adaptive and survival mechanism. The CrdRS TCS is a less-well-investigated system, the name for which is based on its role as a regulator of a copper resistance determinant, CrdA (17). In the gastric environment, H. pylori must maintain proper metal ion metabolism, as these ions are involved in a variety of cellular processes essential for cell growth, and an imbalance can create stress for H. pylori (25). Copper ions specifically play an important role in bacterial homeostasis as cofactors for electron transport, oxidase, and hydroxylases (17).

A 2015 study by Hung and colleagues demonstrated that CrdRS allows H. pylori to mitigate nitrosative stress *in vitro* (12). The results of their study showed a H. pylori *ΔcrdS* mutant, but neither *ΔflgS* nor *ΔarsS*, exhibited significantly reduced viability under NO stress. Through global transcriptome analysis using DNA microarray technology, those investigators identified 145 differentially expressed genes between sham and NO-treated wild-type H. pylori strain 26695. The functions of these genes are involved in cell envelope function, transport and binding, cellular processes, and DNA metabolism, but many of the identified regulated genes have unknown functions.

In the current study, we sought to expand upon the previous investigations of CrdRS-mediated gene expression using RNA-sequencing (RNA-Seq) technology to gain further insights into the CrdRS regulon. The transcriptomic study of Hung et al. (12) focused on differential transcription in the presence of NO, whereas the current study analyzed transcription of CrdRS deletion mutants under standard *in vitro* culture conditions. This expanded delineation of genes affected by CrdRS may facilitate a better understanding of the nature of the requirement for this TCS for H. pylori infection.

## RESULTS

### Analysis of H. pylori gene regulation mediated via the CrdRS TCS.

We compared gene expression between the control and *crdRS* deletion mutants under our standard *in vitro* culture conditions. Two comparisons tested in our RNA-Seq experimental design were (i) H. pylori 26695 Δ*rdxA* (i.e., control mutant) versus H. pylori 26695 Δ*rdx*A Δ*crdS* (i.e., Δ*crdS*), and (ii) H. pylori 26695 Δ*rdx*A versus H. pylori 26695 Δ*rdx*A Δ*crdR* (i.e., Δ*crdR*). Differentially expressed (DE) genes determined by DESeq2 with *P* values of ≤0.05 and ≥2-fold changes in expression levels in each comparison in at least 2 of 3 biological replicates of either Δ*crdS* or Δ*crdR* mutants were considered putative CrdRS regulon members (Table 1). This analysis identified 40 DE genes in one or both mutants. We thus hypothesized them to be members of the CrdRS TCS regulon. Several DE genes were located together in operons: HP0073-HP0072 (*ureAB*), HP0486-HP0487 (*hofCD*), and HP0695-HP0696 (*acxAB*). Although the adjacent genes *HP0463* (encoding a DNA methyltransferase) and *HP0462* (encoding a restriction endonuclease, specificity subunit) are not predicted to be part of the same operon (26), the encoded proteins are predicted to be part of the same type I restriction-modification system (27). It is notable that 20% (8/40) of the DE genes identified encode outer membrane proteins. Interestingly, in a comparison of this putative regulon and the CrdRS regulon members established by Hung and colleagues in 2015 (12), only three genes were shared: *flaB* and the hypothetical protein-encoding genes *HP1120* and *HP1288*. It is notable that *crdA*, encoding a copper resistance determinant protein, was identified as a CrdRS regulon member in the studies of Hung and colleagues (12) as well as by Waidner et al. (17); however, it was not identified as a DE gene in the RNA-Seq analyses of the current study.

**TABLE 1:** Characterization of the 40-gene putative CrdRS TCS regulon determined by RNA-Seq^*a*^

Identification			No. of biological replicates	Δ*crdS*		Δ*crdR*	
RefSeq identification	Locus tag	Gene function		Log_2_ fold change	*P* value^*b*^	Log_2_ fold change	*P* value^*b*^
HP_RS00135	HP0025	*hopD*	3 Δ*crdS*	−2.28	**	♦	ns
HP_RS02410	HP0487	*hofD*	3 Δ*crdS*	−1.67	**	♦	ns
HP_RS04865	HP0991	Hypothetical protein	3 Δ*crdS*	−1.51	**	♦	ns
HP_RS02290	HP0463	SAM-dependent DNA methyltransferase	2 Δ*crdS*	−2.18	***	♦	ns
HP_RS00585	HP0115	*flaB*	2 Δ*crdS*	1.53	**	♦	ns
HP_RS02025	HPt14	tRNA-Pro	2 Δ*crdS*	−1.55	**	♦	ns
HP_RS05990	HP1212	ATP synthase subunit C	2 Δ*crdS*	0.15	***	♦	ns
HP_RS03405	HP0696	*acxB*	2 Δ*crdS*	1.35	**	♦	ns
HP_RS01240	HPt09	tRNA-Ala	2 Δ*crdS*	1.58	**	♦	ns
HP_RS05500	HP1120	Hypothetical protein	2 Δ*crdS*	1.41	**	♦	ns
HP_RS02120	HPr01	23S ribosomal RNA	2 Δ*crdS*	3.67	**	♦	ns
HP_RS03300	HP0671	*horF*	2 Δ*crdS*	−1.41	***	♦	ns
HP_RS03345	HP0682	Hypothetical protein	2 Δ*crdS*	−0.01	**	♦	ns
HP_RS05290	HP1076	Flagellar FLiS export cochaperone	2 Δ*crdS*	1.38	*	♦	ns
HP_RS00370	HP0073	*ureA*	2 Δ*crdS*	1.51	**	♦	ns
HP_RS06355	HP1288	Hypothetical protein	2 Δ*crdS*	−0.07	**	♦	ns
HP_RS00365	HP0072	*ureB*	2 Δ*crdS*	1.84	**	♦	ns
HP_RS02020	HP0410	*hpaA2*	2 Δ*crdS*	−1.29	**	♦	ns
HP_RS02405	HP0486	*hofC*	2 Δ*crdS*	−1.18	**	♦	ns
HP_RS03400	HP0695	*acxA*	2 Δ*crdS*	1.20	**	♦	ns
HP_RS03445	HP0706	*hopE*	2 Δ*crdS*	−1.15	**	♦	ns
HP_RS06740	HP1364	*crdS*	2 Δ*crdS*, 2 Δ*crdR*	−4.13	***	1.60	**
HP_RS07130	HP1440	Hypothetical protein	2 Δ*crdS*, 2 Δ*crdR*	1.57	**	1.40	**
HP_RS06745	HP1365	*crdR*	2 Δ*crdR*		ns	−5.02	***
HP_RS03515	HP0722	Outer membrane beta-barrel protein	2 Δ*crdR*	♦	ns	−2.27	***
HP_RS02935	HP_RS02935	*rnpB*	2 Δ*crdR*	♦	ns	1.52	**
HP_RS00060	HP0009	*hopZ*	2 Δ*crdR*	♦	ns	−1.76	**
HP_RS02285	HP0462	Restriction endonuclease subunit S	2 Δ*crdR*	♦	ns	−2.38	*
HP_RS07355	HP1485	YigZ family protein	2 Δ*crdR*	♦	ns	−1.25	*
HP_RS00470	HP0092	DNA methylase N-4	2 Δ*crdR*	♦	ns	−2.05	***
HP_RS04725	HP0966	GTPase	2 Δ*crdR*	♦	ns	−1.77	**
HP_RS04110	HP0842	Hypothetical protein	2 Δ*crdR*	♦	ns	−2.07	*
HP_RS01450	HP0294	*amiE*	2 Δ*crdR*	♦	ns	1.90	***
HP_RS01940	HP0394	Calcineurin-like phosphoesterase	2 Δ*crdR*	♦	ns	−1.94	***
HP_RS00465	HP0091	DpnII restriction endonuclease	2 Δ*crdR*	♦	ns	−1.85	***
HP_RS03520	HP0723	*ansA*	2 Δ*crdR*	♦	ns	1.52	**
HP_RS07865	HP1589	Ubiquinol-cytochrome *c* chaperone	2 Δ*crdR*	♦	ns	−1.98	**
HP_RS07745	HP1565	*mrdA*	2 Δ*crdR*	♦	ns	−1.46	*
HP_RS05800	HPt28	tRNA-Met	2 Δ*crdR*	♦	ns	−2.81	***
HP_RS02845	HP0578	LTA synthase family protein	2 Δ*crdR*	♦	ns	−2.34	***

a The comparisons were (i) H. pylori 26695 Δ*rdxA* versus H. pylori 26695 Δ*rdxA* Δ*crdS* (Δ*crdS* average) and (ii) H. pylori 26695 Δ*rdxA* versus H. pylori 26695 Δ*rdxA* Δ*crdR* (Δ*crdR* average). ♦, non-DE gene.

b DESeq2 was used to determine significance between the control and the mutants: ***, *P* ≤ 0.001; **, *P* ≤ 0.01; *, *P* ≤ 0.05; ns, *P* > 0.05.

**FIG 1:**
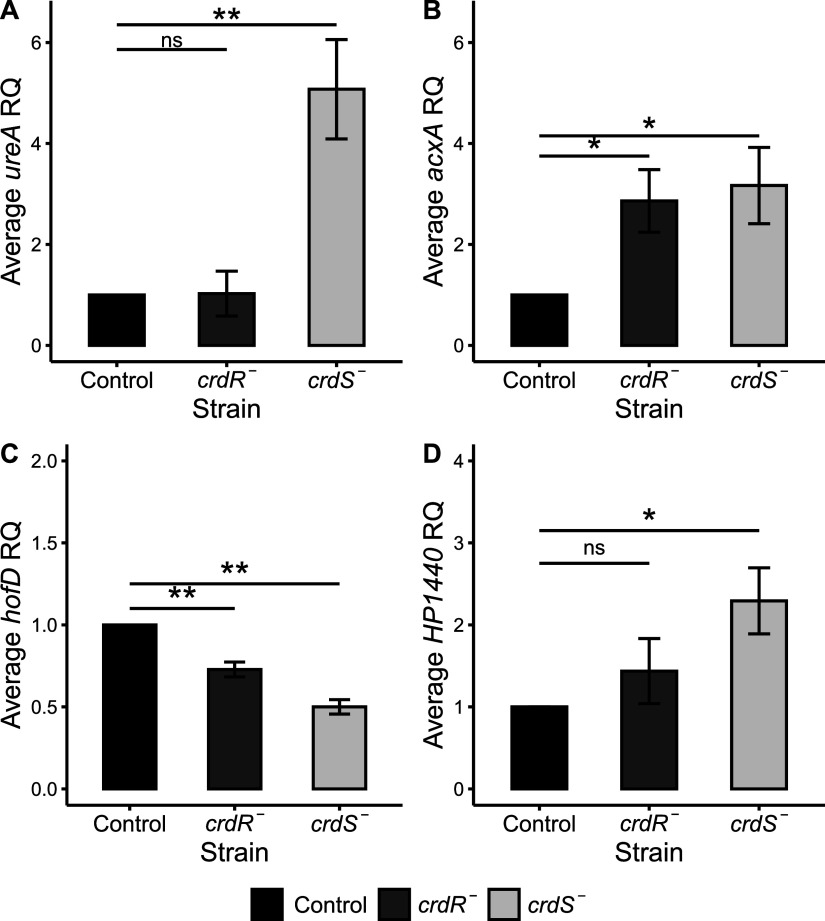
*ureA*, *acxA*, *hofD*, and the H. pylori-specific gene *HP1440* are controlled by the CrdRS TCS. RT-qPCR was used in various H. pylori mutants to quantify mRNA of selected regulon genes as relative quantity (RQ) levels pooled from three biological replicates, each done in technical triplicate, of select regulon genes. Charts show each mutant strain compared to the control H. pylori *rdxA* mutant, where the control was set to 1 and RQ of each deletion mutant (*crdS* and *crdR*) is shown for *ureA* (A), *acxA* (B), *hofD* (C), and *HP1440* (D). For each gene, an analysis of variance (ANOVA) was performed to determine significance between the control and the mutants, followed by a Tukey honestly significant difference (HSD) test. **, *P ≤ *0.01; *, *P ≤ *0.05; ns, *P > *0.05. Several other genes identified as putative CrdRS regulon members via RNA-Seq (Table 1) were also verified by RT-qPCR and are shown in Table S2 in the supplemental material.

We next selected an 11-gene subset of these 40 genes and subjected each to validation via reverse transcriptase quantitative PCR (RT-qPCR). These 11 genes were selected to represent a diverse array of predicted functions related to acid acclimation, outer membrane function, flagellar proteins, acetone metabolism, or proteins with no known function. As in RNA-Seq analyses, we considered genes with a ≥2-fold change in expression levels compared to the control H. pylori mutant in at least 2 out of 3 biological replicates of the RT-qPCR as being regulated by the CrdRS TCS. The RT-qPCR and RNA-Seq data revealed similar patterns and significant differential expression, as did RNA-Seq for 7 of the 11 tested genes when we compared H. pylori 26695 Δ*rdxA* (control) and the Δ*crdS* mutant: *ureA*, *acxA*, *acxB*, *hofC*, *hofD*, *horF*, and *HP1440* (Fig. 1; see also Table S2 in the supplemental material). While the expression of the remaining 29 DE genes suggested by our RNA-Seq analyses have not been assayed in this manner, we speculate that many of the genes listed in Table 1 are also novel members of the CrdRS TCS regulon.

Figure 1 shows the results for four of the seven genes confirmed to be differentially expressed in the CrdRS deletion mutants under the standard *in vitro* growth conditions of this study. The expression of the urease enzyme subunit A gene, *ureA*, was significantly affected by the loss of the histidine kinase sensory portion of the CrdRS TCS, as seen by an increase in *ureA* expression in the H. pylori Δ*crdS* mutant compared to the control mutant (Fig. 1A). Interestingly, *ureA* mRNA levels were unaffected by loss of CrdR. The cotranscribed gene, *ureB*, showed a similar increase in expression in the Δ*crdS* mutant and was also unaffected in the Δ*crdR* mutant (Table 1). The acetone carboxylase subunit A gene, *acxA*, was significantly affected by the loss of both the histidine kinase CrdS and the response regulator of the CrdR, as seen by an increase in its expression in both the Δ*crdS* and Δ*crdR* mutants compared to the control (Fig. 1B). Expression of the outer membrane protein-encoding gene *hofD* was significantly decreased by the loss of either of the CrdRS TCS components, but only the loss of the histidine kinase sensory portion resulted in a ≥2-fold difference (Fig. 1C). Expression of *HP1440*, encoding an H. pylori-specific protein of unknown function, was also affected by the loss of both the histidine kinase sensory portion and the response regulator of the CrdRS TCS; however, it exhibited a >2-fold increase in mRNA due to the loss of the histidine kinase sensor only (Fig. 1D).

While we speculate that these results suggest regulation of these genes by the CrdRS TCS, both the deletion Δ*crdS* and Δ*crdR* mutants harbored large DNA deletions that may have polar effects on the expression of genes reported to be downstream of *crdRS* in an operon (26). To address polarity concerns and further evaluate the role of the CrdRS TCS in gene regulation, we next created novel CrdRS mutants that we predicted to be incapable of phosphorylation (CrdS H173A) or phospho-relay (CrdR D53A) and therefore unable to function in signal transduction.

### Analysis of H. pylori gene regulation in novel phospho-incompetent H. pylori *crdRS* mutants.

**FIG 2:**
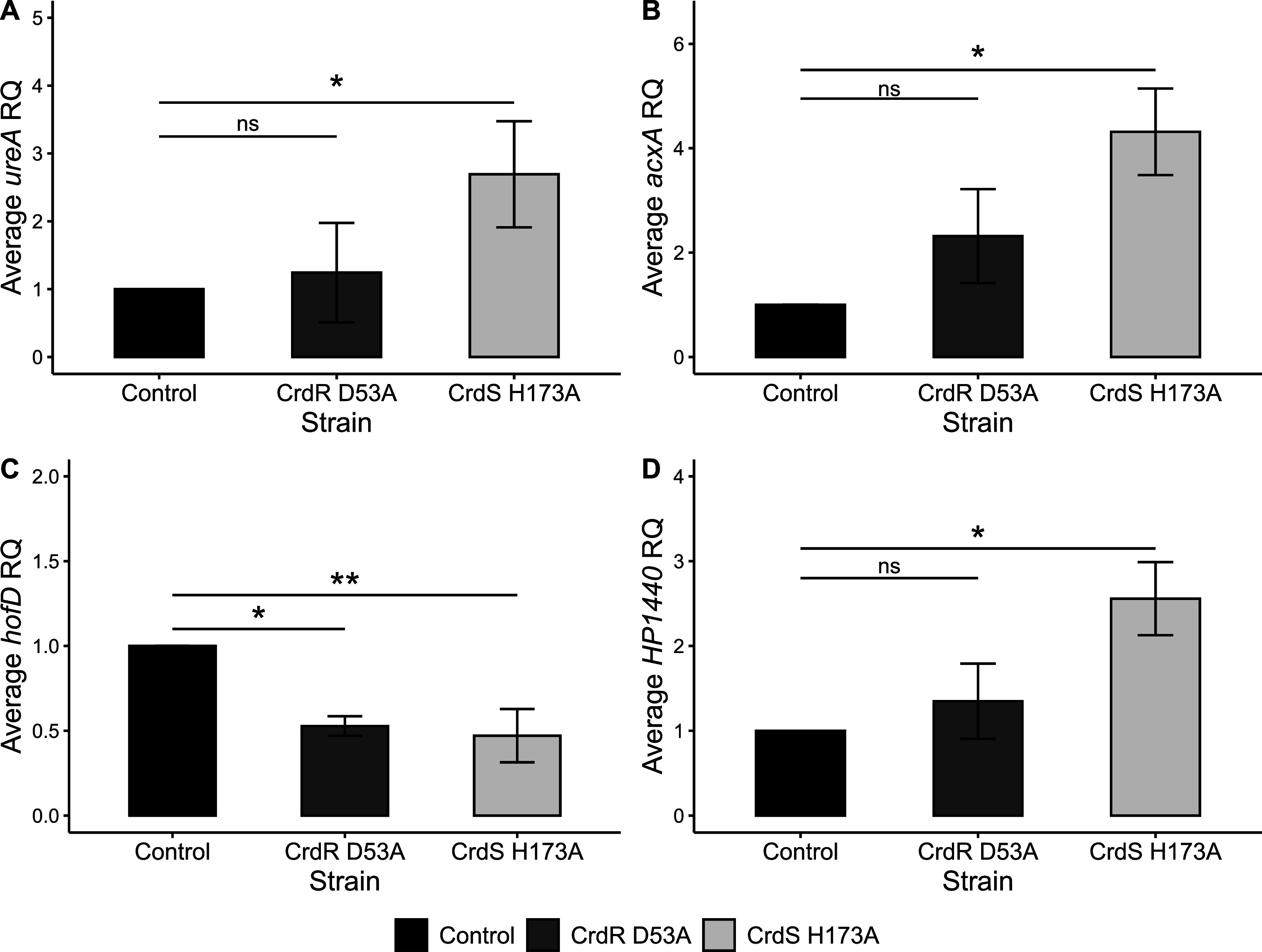
*ureA*, *acxA*, *hofD*, and the H. pylori-specific gene *HP1440* are CrdRS TCS regulon members. RT-qPCR was used in various H. pylori mutants to measure average RQ levels of mRNA based on three biological replicates of selected regulon genes. In the graphs, each strain under standard *in vitro* culture conditions, i.e., mid logarithmic growth phase and no added stimulus, is shown, where the control was set to 1 and expression of each missense mutant is shown. (A) *ureA*; (B) *acxA*; (C) *hofD*; (D) *HP1440*. For each gene, an ANOVA was completed to determine significance between the control and each mutant, followed by a Tukey HSD test. **, *P ≤ *0.01; *, *P ≤ *0.05; ns, *P > *0.05. Two genes, *hofC* and *horF*, which were DE genes in RNA-Seq analyses of Δ*crdS* mutant (Table 1) and significantly affected in either or both Δ*crdS* Δ*crdR* mutants in RT-qPCR analyses (Table S2), were not significantly altered in transcription in the CrdS H173A or the CrdR D53A mutants (Table S3).

We next selected six putative CrdRS regulon genes (*ureA*, *acxA*, *hofC*, *hofD*, *horF*, and *HP1440*) for further RT-qPCR analyses using the novel phospho-incompetent H. pylori 26695 *crdRS* mutants. Both of these novel mutants, H. pylori 26695 Δ*rdxA* CrdR D53A and H. pylori 26695 Δ*rdx*A CrdS H173A, possessed missense mutations rendering them phospho-incompetent due to the loss of the phospho-accepting amino acids aspartic acid and histidine. RT-qPCR mRNA assays were performed to quantify differences in gene expression between the control and CrdRS missense mutants. We considered those genes with ≥2-fold changes in mRNA levels between each mutant compared to the control mutant in at least 2 out of 3 biological replicates as affected by the CrdRS TCS. A comparison of the RT-qPCR expression levels of these genes in the phospho-incompetent *crdR* and *crdS* mutants with those of the *crdRS* deletion mutants is shown in Fig. 2 and Table S3. Significant differences between H. pylori 26695 Δ*rdxA* (control) and the isogenic CrdS H173A mutant were found for 4 genes: *ureA*, *acxA*, *hofD*, and *HP1440*. Additionally, a significant difference between H. pylori 26695 Δ*rdxA* (control) and the isogenic CrdR D53A or CrdS H173A mutants was found for *acxA* and *hofD* (Fig. 2). Thus, *ureA*, *acxA*, *hofD*, and *HP1440* were consistently dysregulated in the absence of a complete CrdRS TCS, as assayed by RNA-Seq (Table 1) and RT-qPCR in both *crdRS* deletion (Fig. 1) and phospho-incompetent mutants (Fig. 2). The expression of the outer membrane protein-encoding genes (i.e., *horF* and *hofC*) in the CrdS H173A and CrdR D53A mutants were neither 2-fold dysregulated nor statistically significant (Table S3).

Figure 2 illustrates the differences in mRNA levels of *ureA*, *acxA*, *hofD*, and *HP1440* between the H. pylori *crdRS* phospho-incompetent mutants and those in the control mutant. Under standard *in vitro* culture conditions, *ureA* expression was not significantly affected by the phospho-incompetence of the CrdR D53A mutant but was significantly affected in the H. pylori CrdS H173A mutant (Fig. 2A). The absence of an effect on *ureA* expression in the CrdR D53A mutant mirrored the effect demonstrated in the Δ*crdR* mutant (compare Fig. 1A and Fig. 2A). While expression of *acxA* was not significantly affected by the H. pylori 26695 CrdR with a D-to-A change at position 53 (D53A) (despite a >2-fold increase in mRNA), a significant increase of >4-fold (*P* < 0.5) in *acxA* mRNA transcript levels was observed in the isogenic CrdS H173A mutant, as indicated by an increase in *acxA* mRNA expression compared to the control mutant (Fig. 2B). Unlike *ureA* and *acxA*, expression of *hofD* was significantly affected in both the CrdS H173A and CrdR D53A mutants (Fig. 2C). Expression of the H. pylori-specific gene *HP1440* was not significantly affected in the CrdR D53A mutant but was significantly affected in the CrdS H173A mutant, as demonstrated by an increase in expression compared to the control (Fig. 2D).

### Novel CrdRS regulon genes *ureA*, *acxA*, *hofD*, and *HP1440* respond to nitrosative stress.

**FIG 3:**
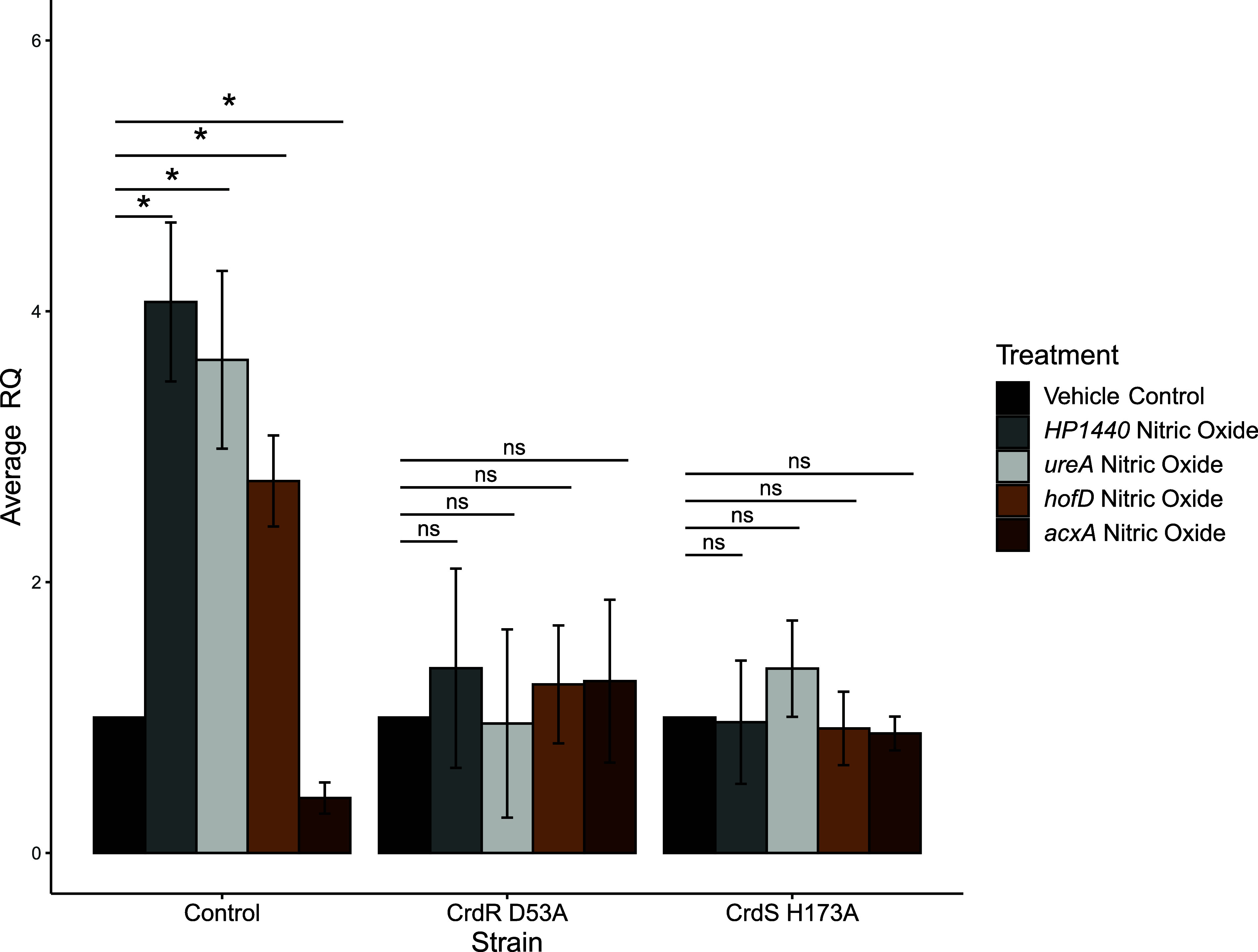
Nitric oxide regulates *HP1440*, *ureA*, *hofD*, and *acxA* expression via the CrdRS TCS. RT-qPCR was used to measure RQ of mRNA under 10 μM nitric oxide and vehicle control conditions for various H. pylori 26695 mutants. The vehicle control condition (PBS) was set to 1, and expression levels of H. pylori isogenic control, and CrdR D53A and CrdS H173A mutants under nitric oxide are shown for *HP1440*, *ureA*, *hofD*, and *acxA*. For each strain, a Student one-tailed *t* test was used to determine significance between the vehicle control and nitric oxide condition. **, *P ≤ *0.01; *, *P ≤ *0.05; ns, *P > *0.05.

As the CrdRS TCS of H. pylori has been demonstrated to respond to NO (11), yet *ureA*, *acxA*, *hofD*, and *HP1440* were not reported among NO-controlled genes, we next evaluated these genes under NO stress conditions in the control H. pylori mutant strain as well as the two CrdRS phospho-incompetent mutants. All four genes had ≥2-fold expression differences assayed after a 4-h, 40 μM NO exposure. In the control strain, genes *HP1440*, *ureA*, and *hofD* were induced and *acxA* was repressed by NO challenge to a significant degree compared to the vehicle control (phosphate-buffered saline [PBS]) (Fig. 3). To confirm that this change in gene expression in response to *in vitro* NO stress was mediated by the CrdRS TCS, the expression of each gene was also evaluated in the two CrdRS phospho-incompetent mutants in the presence and absence of 40 μM NO. For all 4 genes, there were no significant differences in the expression between the mutants under vehicle control treatment and nitric oxide treatment (Fig. 3). This indicated that these newly identified CrdRS TCS regulon genes are also responsive to NO stress in a CrdRS-dependent fashion. This result also confirmed our novel H. pylori CrdR D53A and CrdS H173A mutants as defective in NO signal transduction.

**FIG 4:**
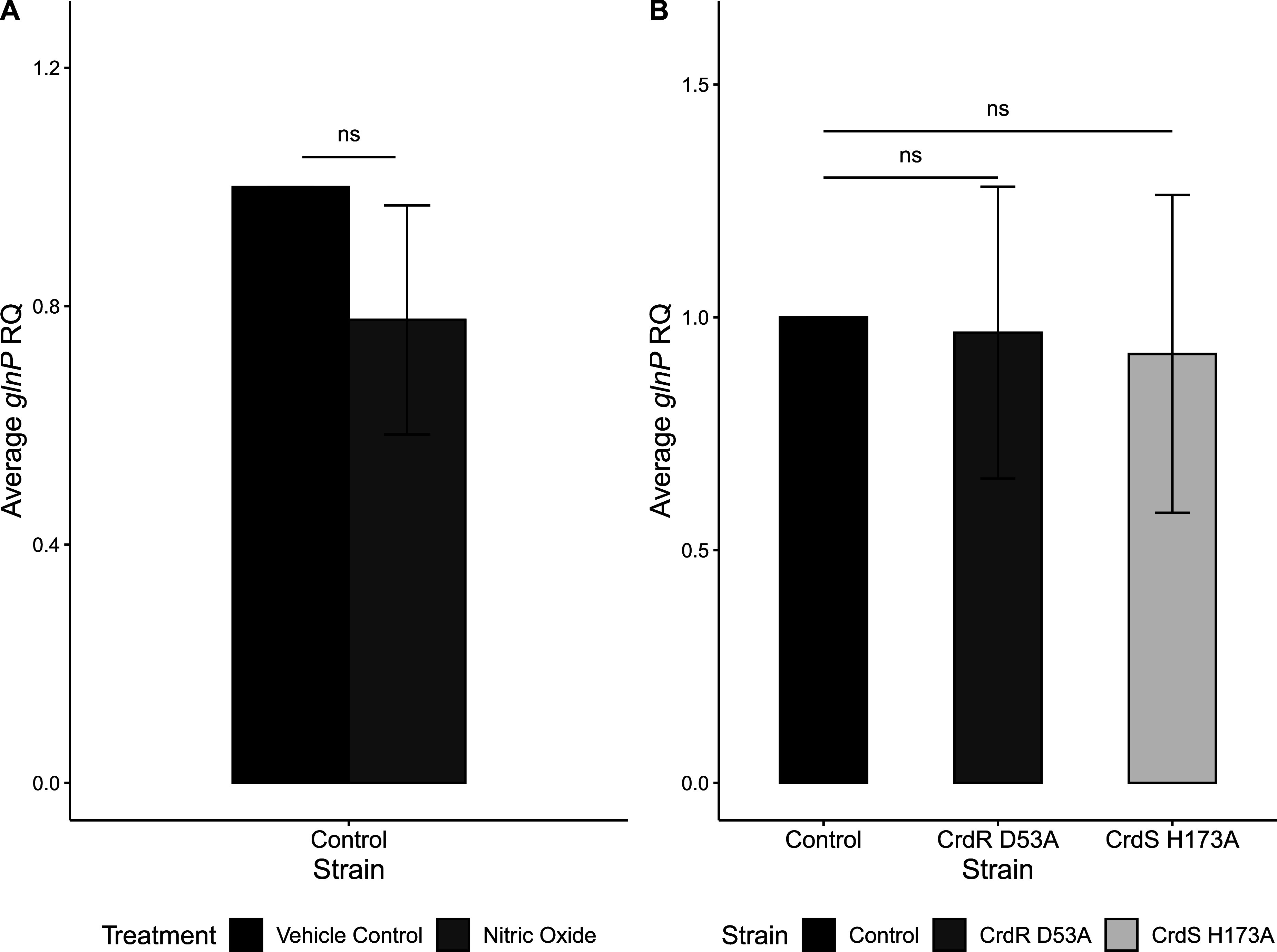
Nitric oxide does not regulate *glnP* expression via the CrdRS TCS. RT-qPCR was used to measure RQ of *glnP* mRNA under 10 μM nitric oxide and vehicle control (PBS) conditions for various H. pylori 26695 mutants. (A) The control mutant under vehicle control conditions was set to 1. Expression of *glnP* under nitric oxide treatment was compared to the control under vehicle control treatment. (B) The control mutant was set to 1, and average RQ of *glnP* mRNA in each phospho-incompetent strain is shown. In graph A, a Student’s one-tailed *t* test was used to determine significance between the vehicle control and nitric oxide condition. For graph B, an ANOVA was performed to determine significance between the control and the mutants, followed by a Tukey HSD test. **, *P ≤ *0.01; *, *P ≤ *0.05; ns, *P > *0.05.

We designed our NO stress experiments to align as closely as possible with the experimental conditions used by Hung and colleagues (12). Although each of our studies used different technology (microarray versus RNA-Seq) to identify CrdRS-regulated genes, we each further examined mRNA levels via RT-qPCR. Because of the disparity between our CrdRS regulons (despite the use of the same H. pylori strain, 26695), we next examined a gene from their CrdRS regulon that responded to *in vitro* NO challenge in their transcriptomic study and was RT-qPCR confirmed in their study, *glnP* (HP1170). This gene was not identified in the studies described in the current study. We observed no significant differences in expression levels of *glnP* between the vehicle control and 10 μM nitric oxide-treated H. pylori control strain (Fig. 4A). Each CrdRS phospho-incompetent mutant was also evaluated under standard *in vitro* culture conditions to reveal CrdRS TCS regulation patterns in the absence of an exogenously added signal. There were no significant differences in *glnP* expression between the control strain and either phospho-incompetent mutant (Fig. 4B).

## DISCUSSION

The CrdRS target genes identified in the current study play important roles in acetone metabolism, an array of outer membrane proteins, and urease function in H. pylori. Urease activity and many outer membrane proteins are critical for H. pylori colonization and persistent infection of the gastric mucosa (28–31). Acetone carboxylase is important for the creation of central metabolites and is also important for H. pylori survival and pathogenesis (32). Acetone carboxylase and urease genes are regulated by multiple H. pylori TCSs and have additionally been shown to be regulated by some orphan response regulator genes (32–35). In 2009, Wen et al. identified acetone carboxylase (*acxABC*) and three urease structural or accessory genes (*ureA*, *ureB*, and *ureI*) as members of the FlgRS TCS regulon (31). Loh et al. established the urease subunits *ureA* and *ureB*, as well as the acetone carboxylase, as ArsRS regulon members (34, 36). Pflock and colleagues (35) identified both the structural urease subunit genes and the acetone carboxylase subunit genes as differentially expressed in an HP1021 null mutant, suggesting that this orphan response regulator is also involved in the regulon of acetone metabolism in H. pylori (35). Additionally, loss of the acetone carboxylase operon (HP0695 to HP0697) results in a reduced ability of H. pylori to colonize mice (32). The current study demonstrated further regulation of urease and acetone metabolism by yet-another H. pylori TCS: CrdRS. This level of genetic investment in the proper regulation of urease and acetone metabolism probably reflects the crucial role these enzymes play in the biology of this pathogen.

In addition to both urease and acetone carboxylase, we showed by both RNA-Seq and RT-qPCR that *HP1440* belongs to the CrdRS regulon and responds to NO challenge. This monocistronic gene, encoding a protein with no known function, is highly conserved among all sequenced strains of H. pylori. BLAST searches revealed *HP1440* homologs are found in virtually no other bacterial species outside the genus *Helicobacter* (data not shown). Such uniqueness, combined with a potential role in response to host-induced stress, makes *HP1440* an interesting target for further investigation as a potentially specific target for intervention in H. pylori pathogenesis. CrdRS TCS regulation of the expression of genes involved in various known and unknown functions reinforces the importance of this TCS in the colonization and survival of H. pylori demonstrated by Panthel and colleagues (18). It is tempting to speculate that other regulon members play key roles in the infectious biology of H. pylori.

A 2005 study by Waidner et al. (17) demonstrated a role for the CrdRS TCS in copper resistance. Those investigators identified a direct role for CrdR in binding a 21-nucleotide sequence associated with the copper resistance determinant protein CrdA (HP1326). They established a clear role for this TCS in resistance to copper *in vitro*. CrdA was additionally identified as a CrdRS regulon member controlled by NO in a 2015 study by Hung and colleagues (12), but it was not identified in the RNA-Seq analyses of the current study. Hung et al. (12) identified a total of 145 NO-responsive genes in wild-type H. pylori 26695, of which 101 were demonstrated to be regulated via the CrdRS TCS (12). That research group’s approach clearly established the NO sensor as CrdS. While this established a regulon of CrdRS TCS-regulated genes under *in vitro* NO challenge conditions, our study sought to identify genes affected by the loss of the CrdRS TCS under standard *in vitro* culture conditions. We speculated that such an approach would identify novel CrdRS regulon members, if they existed.

Our RNA-Seq screen of H. pylori CrdRS TCS regulation revealed 40 genes putatively controlled by this system under *in vitro* culture conditions. Of these 40 genes, 37 were not previously ascribed to the CrdRS regulon (12). Although it is interesting that this putative regulon shares only three genes with the CrdRS regulon members affected by NO identified in the 2015 study by Hung and colleagues (12), it is perhaps unsurprising. Our experimental strategy, designed to complement and extend the findings of Hung et al. (12), was independent of any added NO signal. Additionally, our study utilized a different technology (RNA-Seq) than that used by Hung and colleagues (DNA microarray).

Considering there were only three shared, confirmed regulon members in our study and Hung and colleagues’ 2015 study, it is noteworthy that the novel genes identified in the current study as CrdRS regulon members were also determined to be regulated by NO. Furthermore, despite being identified as a NO-regulated gene via the CrdRS TCS by Hung and colleagues in 2015, *glnP* was neither NO regulated nor CrdRS TCS regulated in the present study. Despite our findings, we do not dispute the results of Hung and colleagues (12). The differences in the results of our respective studies likely suggest an important role of the technology, laboratory conditions, and *in vitro* passage levels of the H. pylori 26695 strains used to establish CrdRS regulons in our respective labs.

A curious feature of the data presented in the current study is that often the deletion of one component of CrdRS, either the sensory histidine kinase or the response regulator, affected regulon member expression, but in only a few cases did the ablation of both components affect regulon gene expression. For example, *ureA* mRNA levels were significantly increased in the Δ*crdS* mutant as assessed by both RNA-Seq and RT-qPCR, but *ureA* mRNA was not significantly affected by deletion of *crdR* (Table 1 and Fig. 1A). This result was corroborated by the use of phospho-incompetent CrdS H174A, resulting in a significant increase in *ureA* mRNA. Again, there was no significant effect on *ureA* mRNA in the CrdR D53A mutant (Fig. 2A). Collectively, this could be interpreted as the absence of a role for the response regulator CrdR in *ureA* regulation via this TCS, potentially suggesting cross talk between CrdS and another transcriptional regulator to mediate the increase in *ureA* mRNA. However, the response of *ureA* mRNA to a 10 μM NO challenge demonstrated that the increased *ureA* expression in response to nitrosative stress was dependent on both components of the CrdRS TCS (Fig. 3).

Our studies of CrdRS regulation of expression of the genes encoding the outer membrane protein *hofD* and the acetone carboxylase operon suggest a rather straightforward, canonical model for the role of CrdRS in the expression of these genes. CrdS promotes the transcription of *hofD*, as the deletion of this sensory histidine kinase (Fig. 1) or a H173A phosphorylation-defective mutation (Fig. 2) resulted in a significant decrease in *hofD* mRNA. This suggested CrdR~P is an activator of the *hofCD* operon. Stimulation of the CrdRS system by NO activates the kinase activity of CrdS and thus increases the cellular pool of CrdR~P, and it results in the observed increase in *hofD* mRNA (Fig. 3). Unlike *hofD*, *acxA* mRNA levels were repressed by NO. This was consistent with our data demonstrating that the ablation of CrdS (Fig. 1) or introduction of a mutant allele incapable of phosphorylation (Fig. 2) resulted in increased *acxA* transcription, indicating that CrdR~P functions to repress transcription of the acetone carboxylase operon. When we challenged H. pylori cells with NO *in vitro*, *acxA* transcription was repressed, likely due to the increase in CrdR~P as the result of phosphotransfer from NO-activated CrdS.

Less straightforward is the role of CrdRS in the regulation of expression of urease and the H. pylori-specific gene encoding a protein of unknown function, *HP1440*. Based upon the data presented in the current study, the presence of a functional sensory histidine kinase CrdS represses both *ureA* and *HP1440* transcription, as there was a significant increase in mRNA of both these genes upon deletion of *crdS.* While these results were largely confirmed using the H. pylori CrdS H173A mutant, an absence of a similar upregulation of *ureA* or *HP1440* mRNA levels in either the *crdR* deletion or the a CrdR D53A mutant under standard *in vitro* culture conditions was observed. This could suggest a lack of involvement of CrdR in the regulation of expression of either of these two genetic loci, despite the clear involvement of its cognate partner, CrdS. However, when quantifying both *ureA* and *HP1440* mRNA in NO challenge experiments, it became clear that NO induction of *ureA* and *HP1440* mRNA was dependent upon both cognate members of this TCS. Thus, CrdRS regulation of both *ureA* and *HP1440* is not straightforward, as it appears with the regulation of *hofD* or *acxA* and may involve *cis*-acting sequences of varying CrdR~P affinities, as seen in the EnvZ-OmpR system of Escherichia coli (37). Alternatively, the regulation of expression of these two H. pylori genes may involve other proteins or even cross talk between TCSs. Collectively, the results indicated that the CrdRS TCS has an important role in regulating H. pylori gene expression.

## MATERIALS AND METHODS

### Bacterial culture.

H. pylori strain 26695 and all isogenic mutants were maintained on 5% sheep blood agar (BBL) plates at 37°C, 5% CO_2_, 95% ambient air and used to inoculate sulfite-free Brucella broth (SFBB) with cholesterol (Gibco BRL) at pH 7 (SFBB/chol/pH 7 broth). The optical density at 600 nm (OD_600_) was used to calculate the inoculum to use to initiate an overnight culture in 5 mL of SFBB/chol/pH 7 broth (OD_600_ = 0.2). Cells were grown overnight at 37°C + 5% CO_2_ to reach an OD_600_ of 1.5 to 2.5. Each culture was then subcultured to identical OD_600_ values and grown to mid-log phase (OD_600_ of 0.8 to 1.2), approximately 6 h. One OD_600_ unit (~10^9^ cells) of the mid-log-phase cultures were then harvested by centrifugation at 6,600 × *g* for 5 min, and cells were resuspended in 1 mL of RNAzol RT (Molecular Research Center Inc.). Once resuspended, each sample was frozen at −80°C for subsequent RNA extraction and cDNA synthesis.

### RNA extraction.

Frozen cultures suspended in RNAzol RT were thawed on ice and subjected to RNA extraction by the manufacturer’s suggested protocol. Briefly, thawed samples were shaken in a Beadrupter (OMNI International Omni Bead Rupter 24) for 45 s. Two microliters of polyacryl carrier (Molecular Research Center Inc.) were then added to aid in precipitation. The samples were cooled on ice, and 400 μL of molecular-grade water was added, followed by vigorous shaking. After a 15-min room temperature incubation, the tubes were centrifuged for 15 min at 12,000 × *g* and 22°C. The supernatant was then removed and transferred into a new tube containing 400 μL of 75% ethanol. The samples were mixed by inversion and incubated at room temperature for 10 min. Samples were centrifuged for 8 min at 12,000 × *g* and 22°C, and the pellet was washed twice with 1 mL of ethanol. RNA pellets were resuspended in 30 to 70 μL of molecular-grade water and immediately quantified using a nanophotometer. Samples were frozen at −80°C for subsequent RNA sequencing and/or cDNA synthesis.

### RNA sequencing.

RNA extracted samples with a concentration of ≥500 ng/mL were then subjected to RNA-Seq by Novogene (Sacramento, CA). Samples were first processed through a quality control step to ensure that each of the samples met the criteria for sequencing. RNA libraries for the target organism and application were then completed for the samples that passed quality control. This was done via rRNA depletion, RNA fragmentation, and reverse transcription of cDNA. Following library creation, the library was processed through a library quality control step. Once the quality was confirmed, a 150-bp paired-end sequence strategy using Illumina Novoseq 6000 was employed to sequence the samples. Data quality control was checked following sequencing, and the gene expression level was measured by transcript abundance and estimated by counting the reads that mapped to genes.

To obtain these counts, the fragments per kilobase of transcript sequence per million base pairs sequenced (FPKM) technique was employed using the FeatureCounts software. FPKM considers the sequencing depth and gene length when counting fragments (38). The counts from FPKM were used as input for DESeq2, which determined differential expression based on a negative binomial generalized linear model. The normalization of DESeq2 calculates the geometric mean for each gene across all samples. The counts for a gene in each sample are then divided by this mean, and the size factor for each sample becomes the median of these ratios in a sample. DESeq2 corrects for library size and RNA composition bias while using shrinkage estimation and fold changes. DESeq2 fits negative binomial generalized linear models for each gene and uses the Wald test for significance testing. It can exclude any outliers and generate a final list of differentially expressed genes for each comparison. These lists were generated and prepared by Novogene.

### cDNA synthesis.

Frozen RNA samples were thawed on ice for cDNA synthesis via an iScript cDNA synthesis kit (Bio-Rad). The total reaction volume was 20 μL, composed of 4 μL 5× reverse transcriptase master mix, 1 μg of RNA. A thermocycler program was run at 25°C for 5 min, 46°C for 20 min, and then 95°C for 1 min with only one cycle. The product was then stored at −20°C until use in RT-qPCR.

### RT-qPCR.

In a MicroAmp EnduraPlate optical 96-well fast clear reaction plate (Applied Biosystems), reactions for the endogenous control (*gyrB*) and target genes were assembled using custom TaqMan gene expression assays labeled with 6-carboxyfluorescein (ThermoFisher) (Table S1); assays were run in triplicate for a reference strain (H. pylori 26695 Δ*rdx*A [39], the control mutant) and one or more additional H. pylori *crdRS* mutants. Each well contained a reaction mixture composed of 10 μL of TaqMan Universal Master Mix II with UNG master mix (Applied Biosystems), 8 μL of molecular-grade water, 1 μL of TaqMan gene expression assay probe (either *gyrB* or target gene-specific probe), and 1 μL of a 1:7 dilution of cDNA. The reaction plate was run in the Applied Biosystems StepOne Plus real-time PCR system for 1 initial cycle (2 min at 50°C, 10 min at 95°C), followed by 40 additional cycles (15 s at 95°C, 1 min at 60°C). Gene expression was determined using the 2^−ΔΔ^*^CT^* method (40).

### Generation of H. pylori *crdRS* deletion mutants.

The *crdR-crdS* locus of H. pylori 26695 was amplified as a 2,498-bp amplicon using primers *crdRS* Fwd A (5′-GCCTTTATGCTTGGCTC-3′) and *crdRS* R1 (5′-CAAAGCATACGAAGAAAACGC-3′) and cloned into pGEM T-Easy (Promega). The resulting plasmid, pCrdRS, was used as a template to create both the *crdR*::CAT-*rdxA* mutant and the subsequent deletions in each of the *crdS* and *crdR* genes.

The subsequent creation of H. pylori mutants in the *crdRS* TCS was done using the counterselection technique described by Loh and colleagues (39). Briefly, for cloning of the CAT-*rdxA* cassette we used a naturally occurring BglII site in the coding sequence of *crdR* from H. pylori strain 26695. The gel-purified pMM674 CAT-*rdxA* BamHI fragment was cloned into the BglII site of *crdR*. The resulting plasmid, pCrdR::CAT-*rdxA*, was then naturally transformed into the H. pylori 26695 *ΔrdxA* strain (36) (metronidizole resistant, chloramphenicol sensitive) with selection for chloramphenicol resistance (10 μg/mL) in the H. pylori 26695 *ΔrdxA* natural transformants. The resulting strain (H. pylori 26695 *ΔrdxA crdR*:CAT-*rdxA*) (39) was used to create subsequent clean deletions individually within *crdS* and *crdR*.

pCrdRS (above) was additionally used as a template in an inverse PCR deletion using 5′-phosphorylated primers iCrdS reverse (5′-ATCCCCATGCGTTTGCATGTGC-3′) and iCrdS forward (5′-GGCGTGTTGGGTTATGGTATAGGG-3′). Inverse PCR using these primers and pCrdRS as template with subsequent DpnI digestion, purification, and self-ligation yielded pΔ*crdS* with a deletion of coding sequence for amino acids 46 through 325 of the *crdS* gene. Deletion of the sequence encoding CrdR amino acids 25 through 200 was performed using inverse PCR of pCrdRS and phosphorylated primers iCrdR-Reverse (5′-ATGCTCCTTAACGCTCTCGC-3′) and iCrdR-Forward (5′-GAAACGCATAAGGGGGTTGGC-3′).

Plasmids thus containing deletions of the coding sequences of *crdS* and *crdR*, pΔcrdS and pΔ*crdR*, respectively, were used in natural transformation of H. pylori 26695 ΔrdxA *crdR*:CAT-*rdxA* (38). Metronidazole-resistant clones, resistant to 7.5 mg metronidazole mL^−1^, were screened by PCR and sequencing to confirm deletion of *crdS* and *crdR*. The resulting mutants were H. pylori 26695 *ΔrdxA ΔcrdS* and H. pylori 26695 *ΔrdxA ΔcrdR*.

### Generation of phospho-incompetent H. pylori *crdRS* mutants.

To create amino acid substitutions in H. pylori 26695 *crdR* and *crdS* to render them incapable of phosphorylation, primers were designed to anneal upstream of *crdR* and downstream of *crdS* to amplify portions of the adjacent sequences as well as full-length *crdR* and *crdS* (5′-GCGTTTTCTTCGTATGCTTTG-3′ and 5′-GCCTTTATGCTTGGCTC-3′). PCR using the designed primers yielded an amplicon of 2,498 bp from H. pylori strain 26695. The amplicon was then cloned into the pGEM-T Easy vector (Promega).

The resulting plasmid was used in site-directed mutagenesis using a QuikChange Lightning Multi site-directed mutagenesis kit (Agilent) and primers introducing H173A and D53A mutations into *crdS* and *crdR*, respectively. The primers, with the mutation bolded and underlined, were *crdS* H173A, 5′ AAA ACA CC ACG **GC**T GAA TTA AAC ACC CCC ATG AGC GC 3′, and *crdR* D53A, 5′ G CGC TTC AAC CTC TTG CTT TTA G**CA** GTG CAA GTG CCT G 3′. Mutant plasmids were confirmed by sequencing and then naturally transformed into H. pylori 26695 Δ*rdxA crdR*::CAT-*rdxA* (39) using metronidazole (7.5 mg/mL) to select potential allelic replacements. Mutants were validated by PCR and sequencing to confirm the missense mutations were incorporated into the H. pylori genomes.

### NO challenge.

Two 36- to 48-h 5% sheep blood agar (BBL) plates of H. pylori cells grown at 37°C, 5% CO_2_ were harvested into 3 mL of SFBB/chol/pH 7 broth. Initial broth cultures in SFBB/chol/pH 7 were inoculated to an OD_600_ of 0.2 and grown overnight at 37°C, CO_2_ to reach an OD_600_ of 1.5 to 2.5.

Each culture was then subcultured and allowed to grow until mid-log phase of growth (OD_600_ of 0.8 to 1.2). The cultures were subjected to a 10 μM NO (spermine NONOate; ThermoFisher) challenge or vehicle control (PBS) treatment for 4 h at 37°C, 5% CO_2_, 150 rpm. Cells were harvested by collecting 1 OD_600_ unit (~10^9^ cells) and centrifugation, and the cells were resuspended in 1 mL of RNAzol RT (Molecular Research Center Inc). Once resuspended, each sample was frozen at −80°C for subsequent RNA extraction and cDNA synthesis.

### Data availability.

The RNA-seq data have been deposited in the GEO database under accession number GSE221309.
