# Supplementary material for: Regulation of Helicobacter pylori Urease and Acetone Carboxylase Genes by Nitric Oxide and the CrdRS Two-Component System
Source: Microbiol Spectr. 2023 Jan 10;11(1):e04633-22. doi: 10.1128/spectrum.04633-22 (PMC9927306; doi:10.1128/spectrum.04633-22)
Supplement: Supplemental file 1 — Tables S1 to S3. Download spectrum.04633-22-s0001.pdf, PDF file, 0.2 MB [file spectrum.04633-22-s0001.pdf]

Supplemental Information

Table S1. **Custom TaqMan gene expression assays used in RT-qPCR.**

Gene Name	Forward Primer	Reverse Primer	Reporter
gyrB	AAAGCCAGAGAGCTTACAAGGAAAA	CGCCCTCCACTAAAAAGATTTCACT	TTGCCTGGAAAATTAG
ureA	AAGACATCACTATCAACGAAGGCAA	CGATTTGAACCGGTCTGTCG	CACGCTAACGGCTTTT
acxA	TCAAACGCTGATACCCACTTCTC	CATCTGGATTCATCCAAGTCCCTTT	ATGGCGCTTATTTTGC
acxB	GGGAGTTTTGTGCGCATTGAT	GCTGTCAGGCCCTAGTTTGA	CCACACAGCCGATCTG
hofC	GTGGTTGTTCCCTATCTATCGTGAA	ACACATAAGGGTGGATCATCAAATTCTTT	CCCGCTTTATGAACCC
hofD	GCGTCAAGCGACTGGTTTT	CCATTAGAGCCTTACCGCTTGTATA	TAACGCCCAAAATACC
hopZ	CGCCTATGTGGAAGAGACGATA	CGGCTTGAGTGCCAAAATGC	ACCGCCCTAAATAACA
hopE	GGCAAGCAAGTTTATGCACCTAAT	TCGTTATCAATAATATCAGCTAACAAATCGCT	CCACACCCCAAGAGAC
horF	GTATTTGGAGCGAACGCTTTAACAT	GTCGCACATGGTTGGTGAAG	ACACCCCACCATTATC
flaB	TGTGGAAGCGAGCTTGGATATTC	CGCCCGTCAATGGAGTGT	AAGGGCGCATTAATTT
HP_1440	GGAATCAAGGGCTTTCGCTTTTAA	TCCTTGCATGATAATAGCTCTCTTCTCT	AACCGCCGATTTTAG
HP_1076	CCATGATTGTGGTTAATCCGTTGGA	CTCAAATAACCTAATAATTCCTTAATTTCGTTTAATTTTTCTTC	TCGCCCACATTTTCCA
glnP	ATCGGTTTGAGCGCTTTAGAGT	CTCATATACCCCCCACCCAAAAA	CCCTAACGCTAAAATC

The reporters have FAM as the fluorophore at the 5’ end and NQR as the quencher at the 3’ end.

Table S2. **Validation of a subset of the CrdRS TCS regulon via RT-qPCR**.

Identification			RNA-Seq				RT-qPCR			
			Δ*crdS* Average		Δ*crdR* Average		Δ*crdS* Average		Δ*crdR* Average	
RefSeq Identification	Locus Tag	Gene Function	Fold change	P-value	Fold change	P-value	Fold change	P-value	Fold change	P-value
HP_RS03405	HP0696	*acxB*	2.54	**	◊	ns	4.69	***	4.98	**
HP_RS00585	HP0115	*flaB*	2.90	**	◊	ns	◊	ns	0.40	**
HP_RS02405	HP0486	*hofC*	0.44	**	◊	ns	0.31	***	◊	ns
HP_RS03445	HP0706	*hopE*	0.45	**	◊	ns	◊	ns	◊	ns
HP_RS00060	HP0009	*hopZ*	◊	ns	0.30	**	◊	ns	◊	ns
HP_RS03300	HP0671	*horF*	0.38	***	◊	ns	0.40	**	0.68	*
HP_RS05290	HP1076	Flagellar FLiS export co-chaperone	2.60	*	◊	ns	◊	ns	◊	ns

Table S2 shows RefSeq identification, locus tag, gene function, average fold change per condition of three biological replicates, and average p-value of each condition. The comparisons were (i) *H. pylori* 26695/Δ*rdx*A (control) vs. *H. pylori* 26695/Δ*rdx*A/Δ*crdS* (**Δ*crdS* Average**) and (ii) *H. pylori* 26695/Δ*rdx*A (control) vs. *H. pylori* 26695/Δ*rdx*A/Δ*crdR* (**Δ*crdR* Average**) for RNA-Seq and RT-qPCR. ◊ represents non-DE. DESeq2 determined

significance between the control and the mutants for RNA-Seq and an ANOVA was performed to determine significance between the control and the mutants, followed by a Tukey HSD test for RT-qPCR (*** = $p \leq 0.001$, ** = $p \leq 0.01$, * = $p \leq 0$ .05, ns = p > 0.05). Additional verifications shown in Figure 1.

Table S3. **Confirmation of the CrdRS TCS target regulon via CrdRS phospho-incompetent missense mutants using RT-qPCR**.

Identification			Deletion Mutants				Phospho-Incompetent Mutants			
			Δ*crdS* Average		Δ*crdR* Average		CrdS H173A		CrdR D53A	
**RefSeq Identification**	**Locus Tag**	**Gene Name**	**Fold change**	**P-value**	**Fold change**	**P-value**	**Fold change**	**P-value**	**Fold change**	**P-value**
HP_RS02405	HP0486	*hofC*	0.31	***	◊	ns	◊	ns	◊	ns
HP_RS03300	HP0671	*horF*	0.40	**	0.68	*	◊	ns	◊	ns

Table S3 shows RefSeq identification, locus tag, gene function, average fold change per condition, and average p-value of each condition. The comparisons were *H. pylori* 26695/Δ*rdxA* (control) vs. (i) *H. pylori* 26695/Δ*rdxA*/Δ*crdS* (**Δ*crdS* Average**), (ii) *H. pylori* 26695/Δ*rdxA* vs. *H. pylori* 26695/Δ*rdxA/*Δ*crdR* (**Δ*crdR* Average**), (iii) *H. pylori* 26695/Δ*rdx*A/CrdS H173A (**CrdS H173A**), and (iv) *H. pylori* 26695/Δ*rdxA*/CrdR D53A (**CrdR D53A**) for three biological replicates of RT-qPCR. An ANOVA was performed to determine significance between the control and each mutant, followed by a Tukey HSD test (*** = $p \leq 0.001$, (** = $p \leq 0.01$, * = $p \leq 0$ .05, ns = p > 0.05). Additional genes shown in Figure 2.
